# Comparative Analysis of Bird Collision Prevention Patterned Samples: Avoidance Performance Under Transparent and Reflective Glass Conditions in Flight Tunnel Tests

**DOI:** 10.3390/ani16213332

**Published:** 2026-10-22

**Authors:** Minyoung Kim, Ryeoeun Youn, Choonghyun Kang, Jong Koo Lee

**Affiliations:** 1 Division of Life Science, Incheon National University, 265 Harmony-ro, Yeonsu-gu, Incheon 22014, Republic of Korea; minykim95@gmail.com (M.K.); maiyoon0303@gmail.com (R.Y.); cndgus1054@gmail.com (C.K.); 2 Bird Collisions Prevention Association, 425 Doksanseong-ro, Osan-si 18105, Republic of Korea; 3 Bio-Resource and Environmental Center, Incheon National University, 119 Academy-ro, Yeonsu-gu, Incheon 22012, Republic of Korea

**Keywords:** bird window collisions, glass reflection, flight tunnel

## Abstract

The transparency and reflectivity of glass are primary factors contributing to bird collisions, as they prevent birds from recognizing glass surfaces as obstacles. However, the effectiveness of bird collision prevention products is primarily evaluated through transparency-based tunnel tests, which do not adequately account for the reflective conditions commonly found on building glass. We developed an indoor flight tunnel system that simulated sky reflections on glass surfaces and compared avoidance responses to three bird collision prevention patterned samples under both transparent and reflective conditions. A total of 507 wild small passerines were tested, each undergoing a single flight trial. All three patterned samples showed lower avoidance rates under reflective than transparent conditions, but the overall effect of test condition on avoidance was not statistically significant. The frequency of invalid flights increased significantly under reflective conditions. These findings suggest that avoidance responses to bird collision prevention products may vary between transparent and reflective test configurations, although the magnitude of this difference remains uncertain. Incorporating reflective conditions alongside conventional transparency-based tests may therefore provide complementary information for evaluating product performance under different optical conditions.

## Simple Summary

Birds frequently collide with glass because they may perceive the surrounding environment seen through or reflected on glass as open space. Bird collision prevention products are used to reduce such collisions, but their performance has mainly been evaluated under conditions that focus on glass transparency. In this study, we used a flight tunnel to test whether birds avoided glass with bird collision prevention products and reproduced sky reflections similar to those seen on building glass. Three patterned samples were compared under transparent and reflective conditions. All three samples showed lower avoidance rates in reflective conditions, but the differences were not statistically significant. Invalid flights occurred more frequently under reflective conditions. These results suggest that reflections may influence how birds respond to bird collision prevention products and should be considered alongside transparency when evaluating products for use on buildings.

## 1. Introduction

Bird collisions are among the most common problems associated with contemporary built environments [1] and represent a significant factor contributing to global declines in bird populations [2]. In the United States alone, it is estimated that between 365 and 988 million birds die each year as a result of collisions with buildings [3]. Furthermore, a recent study found that approximately 60% of birds rescued following collisions with buildings subsequently died, indicating that the actual mortality rate associated with bird collisions may be significantly higher than previously estimated [4]. This issue is not confined to North America but has also been documented in urbanized regions worldwide, including Asia, and is consequently recognized as a significant international biodiversity conservation concern [5,6]. Glass is widely employed in modern building facades to admit natural daylight and establish visual connections with the outdoor environment [7], leading to large glass surfaces that may increase the risk of bird collisions. Consequently, bird collisions remain an important challenge for urban biodiversity conservation [1,3]. Accordingly, recent research has concentrated not only on the development of novel materials and products aimed at reducing collisions [8] but also on the advancement of methodologies for assessing the expected performance of bird collision prevention products in real-world structural applications where such collisions occur [9,10]. Whereas collisions at night are driven primarily by attraction to artificial light [3], collisions in daylight occur because the transparency and reflectivity of glass prevent birds from perceiving it as a barrier [1,11]. The present study addresses this daytime, glass-related component of collision risk, which is the target of bird collision prevention products.

Bird collisions with glass occur because its transparency and reflectiveness prevent birds from perceiving the glass surface as a physical barrier [1]. Transparency allows the sky or vegetation visible through the glass to be perceived as a continuous space, which birds may interpret as a navigable flight path. In contrast, reflections of the surrounding environment on glass surfaces can generate visual patterns that mimic natural habitats, thereby increasing the risk of bird collisions. These reflective conditions are considered a principal factor contributing to the frequent occurrence of bird collisions with glass buildings [12]. Specifically, highly reflective materials, such as mirrored glass, can produce intense reflections regardless of sunlight intensity or direction, thereby potentially posing a significantly elevated risk of collision [7]. To reduce bird collisions, patterns or materials are applied to glass at regular intervals to enhance its visibility to birds. These patterns are distinctly visible on the glass, assisting birds in recognizing it as a barrier and thus preventing collisions [13,14]. Pattern spacing is considered a critical design parameter affecting the efficacy of avoidance. The “2 × 4 rule,” originally proposed by Klem [14], recommends a maximum spacing of 5 cm vertically and 10 cm horizontally between pattern elements. More recently, the more conservative “2 × 2 rule,” which restricts spacing to 5 cm or less in both dimensions, has been recommended to accommodate smaller bird species [1]. However, the effectiveness of a pattern is influenced not only by spacing but also by visual attributes such as the size, arrangement, and orientation of its elements, which can affect avoidance responses [14,15]. Therefore, to effectively reduce bird collisions, the applied patterns must not only adhere to appropriate spacing criteria but also be sufficiently distinguishable from the surrounding background to function as visible indicators of the glass surface [13]. Under transparent conditions, bird collision prevention patterns are displayed on the glass surface, allowing the visual background to remain visible behind it. In contrast, under reflective conditions, prevention patterns are seen against reflected environmental elements, such as the sky or vegetation [15,16]. The effectiveness of visual markers depends on their visibility against the glass background; accordingly, this visual overlap may reduce the contrast or distinctiveness of the prevention patterns, potentially compromising their function as visual cues indicating the presence of a physical barrier [15,16]. Simultaneously, reflections of the sky or vegetation may be interpreted as extensions of navigable space, thereby further complicating the recognition of the glass surface as a physical barrier. UV-reflective patterns, which have garnered increasing research interest in recent years, may be effective only for certain bird species, as sensitivity to ultraviolet wavelengths varies among them [17]. Bird collisions are therefore associated not only with the optical properties of glass but also with inherent limitations of the avian visual system. The avian visual system is adapted to provide a wide peripheral field of view, whereas the frontal binocular field remains relatively restricted in many species. In addition, behaviors such as directing visual attention laterally or downward during flight may temporarily restrict visual information in the direction of travel [18], potentially reducing the ability of birds to recognize artificial structures as obstacles. Bird collisions may also result from the fact that artificial structures lie outside the evolutionary experience of birds, limiting their learned ability to anticipate and avoid such obstacles [19]. 

Bird collision prevention products are commonly evaluated using standardized flight-tunnel tests that compare birds’ avoidance responses to different treatments [15,20]. However, most existing tunnel tests primarily simulate collision scenarios involving glass transparency by presenting a background behind the glass [13,15]. While this approach is appropriate for evaluating whether birds perceive the environment visible through glass as a navigable flight path, it does not comprehensively address the reflective conditions frequently encountered in urban glass architecture. Product performance may vary depending on placement and the optical properties of the glass, as externally applied window films have been found to be more effective than identical treatments applied internally; this difference is attributed to reflection from the untreated exterior surface [18]. Reflection itself can also alter collision responses, with stronger reflection associated with approximately four-fold fewer collisions under bright midday conditions but a non-significant doubling under morning conditions [21]. Thus, the effects of reflection appear to be context-dependent; however, quantitative comparisons of identical bird collision prevention products under directly comparable transparent and reflective conditions remain limited. Recent studies have emphasized the need to assess how well tunnel test results reflect product performance under real-world collision conditions [1]. From this perspective, incorporating reflective conditions commonly encountered in actual built environments into product evaluations may help assess the applicability of conventional tunnel tests across a broader range of glass conditions [1,15].

This study presents the development of a tunnel testing system designed to replicate reflective conditions similar to those found on the glass surfaces of actual buildings. We compared avoidance rates and the occurrence of invalid flights under transparent and reflective conditions to determine whether the responses observed in conventional transparency-based tests remain consistent under reflective conditions. 

## 2. Materials and Methods

### 2.1. Design of Tunnel Test System

**Figure 1:**
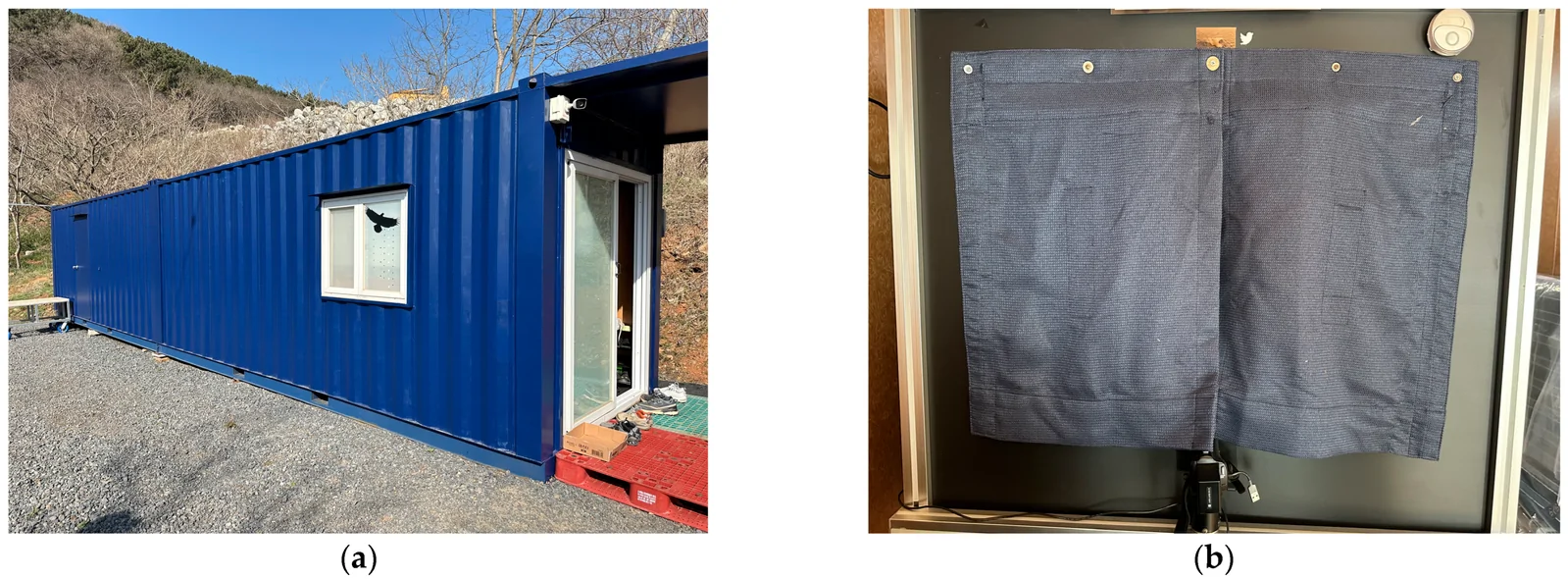
Indoor flight tunnel testing facility. (**a**) Exterior view of the light-shielded container housing the flight tunnel; (**b**) starting end of the flight tunnel showing the bird access opening and video camera.

This study used an indoor flight tunnel system to compare the avoidance responses elicited by the same bird collision prevention patterned samples under transparent and reflective conditions. The experimental facility was located on Socheongdo Island, Ongjin-gun, Incheon, Republic of Korea, and bird collision tunnel tests were conducted during the spring migration periods from April to June in 2025 and 2026. The tests under transparent conditions were conducted in 2025. After developing the reflective test configuration in 2026, the same three patterned samples were assessed under reflective conditions during the spring migration period. The flight tunnel was based on the conventional outdoor tunnel design proposed by the American Bird Conservancy (ABC) [11]. However, to minimize the effects of external weather conditions, solar position, and changes in natural light on the test environment, the tunnel was installed inside a light-shielded steel container measuring 2 m × 2 m × 12 m (Figure 1a). During testing, external natural light was blocked, and artificial lighting was used to maintain consistent illumination of the test glass and background. Full-spectrum LEDs with a continuous spectrum of approximately 400–700 nm, corresponding to the visible range of natural daylight, were used as the light source [19]. The flight tunnel consisted of a straight passage measuring 7.4 m in length, 1.0 m in width, and 1.0 m in height. The interior of the tunnel was kept dark to direct birds toward the glass at the terminal end while minimizing visual distractions from the side walls and surrounding structures. The tunnel structure was constructed using dark, non-reflective materials to minimize unwanted light reflections and visual interference from surfaces other than the test glass. At the entrance of the tunnel, an access opening was installed for introducing birds, and a video camera was mounted below this opening, directed toward the terminal end of the tunnel, capturing both glass panes within the recorded image (Figure 1b). At the terminal end of the tunnel, a mist net (mesh size: 16 mm × 16 mm; Ecotone) was installed to prevent birds from directly colliding with the test glass during flight. The glass panes were secured to a frame positioned 15 cm behind the mist net. The frame was designed to hold two glass panes, each measuring 50 cm × 100 cm, side by side. One pane served as the treatment glass to which a bird collision prevention product was applied, whereas the other served as an untreated control glass. The two panes were installed in the same plane with identical height, inclination, and approach distance. Before testing the patterned samples, a preliminary test using two identical untreated glass panes was conducted to assess potential left–right preference. Of the 80 valid flights, 39 were directed toward the left pane and 41 toward the right pane, with no statistically significant side preference (exact binomial test, *p* = 0.911). The positions of the treatment and control panes were determined before testing and remained fixed throughout all trials for each sample. Under both transparent and reflective conditions, the treatment pane was positioned on the left and the control pane on the right for all three samples. Glass panes of the same dimensions and configuration were used under both transparent and reflective conditions, and the placement and application area of each bird collision prevention product were kept identical between the two conditions.

Under the transparent condition, the test environment was configured to reproduce the conditions used in conventional bird flight tunnel tests. A background panel displaying a sky image was positioned 30 cm behind the test glass, allowing birds to view a bright background through the glass. Full-spectrum LED lights (Samsung horticulture LEDs, Samsung Electronics Co., Ltd., Suwon, Republic of Korea) were installed above and below the glass frame. These lights provided a continuous spectrum across the visible range (approximately 400–700 nm), including blue, green, and red wavelengths, to provide lighting conditions similar to natural daylight. UV light sources, which are detectable by some bird species, were also installed to extend the spectral range available in the test environment. The background, lighting direction, and light intensity were kept identical for the treatment and control glass, such that the presence or absence of the bird collision prevention product was the only intended visual difference between the two panes.

**Figure 2:**
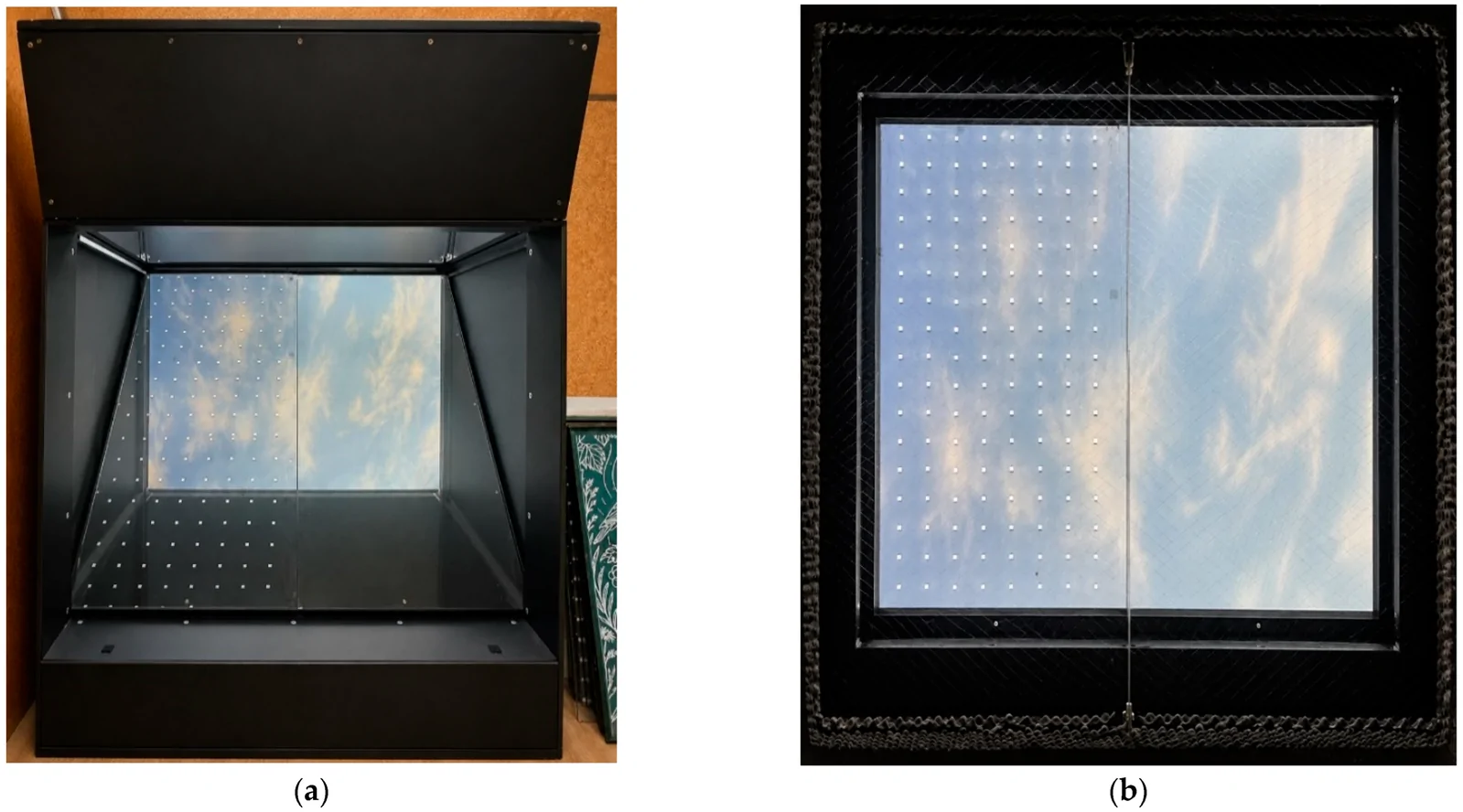
Reflective-condition test setup. (**a**) Interior configuration of the reflective test structure; (**b**) view from the tunnel showing the reflected sky on the treatment and control glass surfaces.

Under the reflective condition, a separate test structure was constructed to reproduce an environment in which the surrounding sky is reflected on the glass surface. The reflective structure consisted of a box-shaped enclosure measuring 1 m × 1 m × 1 m, with two glass panes, each measuring 50 cm × 100 cm, installed side by side. The two panes were positioned on the same inclined plane and fixed at an angle of 45° from the vertical (Figure 2a). A 1 m × 1 m back-illuminated panel displaying a sky image was installed on the ceiling of the reflective structure. The panel was back-illuminated through a diffuser using the same light source as that used under the transparent condition, allowing the sky image on the ceiling panel to be reflected on the surfaces of both glass panes (Figure 2b). The interior surfaces of the reflective structure, including the sides, bottom, and entrance area, were covered with matte black panels. This configuration was intended to minimize reflections of indoor lighting and surrounding structures on the glass surfaces and to maintain the ceiling-mounted sky image as the primary reflected background. To limit the direct entry of external light and maintain consistent reflective conditions within the box, the reflective structure was positioned approximately 15 cm from the terminal end of the tunnel using a support attached to the entrance of the structure. The flight distance from the release point to the mist net was identical under both test conditions. This offset also provided a buffer space between approaching birds and the reflective structure. The treatment and control glass panes were positioned side by side on the same inclined plane to maintain identical optical conditions. As in the transparent condition, a bird collision prevention pattern was applied to the treatment glass, whereas the control glass remained untreated. The reflected background and lighting conditions were kept identical for both panes, such that the presence or absence of the bird collision prevention pattern was the only intended visual difference between the treatment and control glass. This configuration was designed to compare avian responses when the visual background was visible through the glass with those observed when it appeared as a reflection on the glass surface.

### 2.2. Tunnel Test Procedure

The same testing procedure was used under both transparent and reflective conditions. After morphological measurements were completed, each bird was placed on the researcher’s hand at the entrance of the tunnel and allowed to initiate flight voluntarily. If the bird initiated flight within 30 s, its flight direction toward either the treatment or control glass was recorded through direct visual observation via an observation aperture located in the upper portion of the screen at the release end of the tunnel, from which both glass panes and their respective treatment and control positions were visible. The directional choice was defined as the first clearly oriented flight toward either glass pane. A flight toward the control glass was classified as C, whereas a flight toward the treatment glass with a bird collision prevention pattern was classified as T. After each trial, the video footage recorded by the camera installed at the tunnel entrance was reviewed to verify the direct-observation classification and to determine the final flight classification. Observers were aware of the treatment and control pane positions during flight classification, and agreement between direct observations and video-based classifications was not quantitatively assessed. Upon completion of each test, the birds were immediately released into the wild through an exit at the terminal end of the tunnel. The avoidance rate (AR) was calculated as the proportion of valid flights directed toward the control glass and was expressed as a percentage to two decimal places. For product scoring, AR values were subsequently rounded to the nearest whole number, corresponding to the tunnel score [22]. Testing of each sample was conducted until a minimum of 80 valid flights had been recorded, in accordance with the standard sample size employed in tunnel testing [22].

Flights were classified as invalid under any of the following circumstances: (1) the bird did not initiate normal flight and landed on the floor or side of the tunnel, or stopped during flight and subsequently resumed flying; (2) the bird did not initiate flight within 30 s while positioned on the researcher’s hand; (3) the bird stopped near the safety net without flying toward either glass pane or repeatedly changed direction between the left and right sides; or (4) the bird moved at an unusually slow speed before reaching either glass pane, such that the flight could not be considered a normal flight response.

These criteria were used to identify trials in which a valid directional choice between the treatment and control panes could not be determined. Invalid flights were excluded from the avoidance-rate analysis and analyzed separately to determine whether their occurrence varied across test conditions.

### 2.3. Test Samples

**Figure 3:**
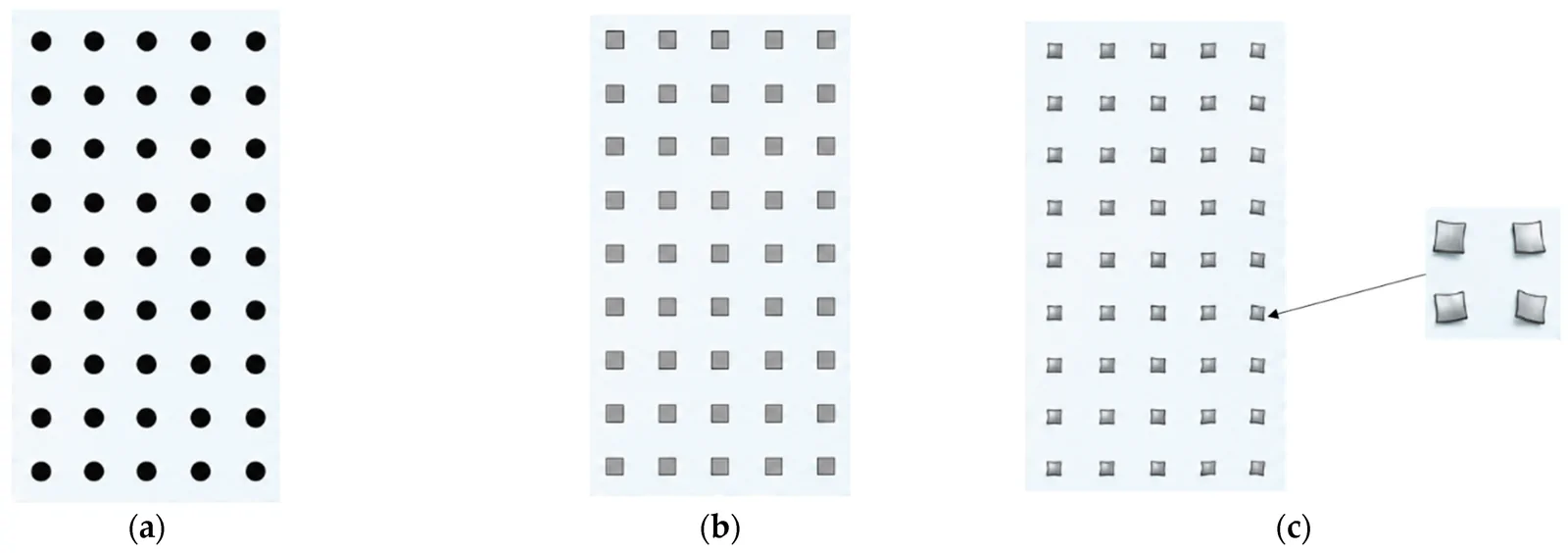
Test patterned samples used in the flight tunnel experiments. (**a**) Black circular pattern (10 mm in diameter); (**b**) silver square pattern (8 mm per side); and (**c**) three-dimensionally oriented silver square pattern (6 mm per side) embedded between two glass panes by lamination. For all samples, the spacing between adjacent pattern elements was 5 mm in both the horizontal and vertical directions.

Three bird collision prevention patterned samples, each featuring distinct pattern configurations, were designed and utilized as test samples in this study. The samples consisted of black circles with a diameter of 10 mm (Figure 3a), silver squares with sides of 8 mm (Figure 3b), and silver squares with sides of 6 mm (Figure 3c). For all samples, the spacing between adjacent pattern elements was maintained at 5 mm in both the horizontal and vertical directions. For Samples a and b, the test and control panes consisted of 3-mm conventional clear glass without additional surface coatings. Individual decals were applied directly to the exterior surface of the test glass facing the approaching birds. No continuous transparent carrier film remained on the glass surface after application. Sample c consisted of two 3-mm clear glass panes laminated together. In the test pane, reflective silver square patterns were embedded between the two glass layers, while the control pane consisted of an identical laminated glass construction without these patterns. Unlike the surface-applied patterns in Samples a and b, the pattern elements in Sample c were oriented at different angles across multiple planes. Consequently, the appearance and intensity of light reflected from the pattern elements varied according to the viewing angle and the direction of the incident light. For each sample, the treatment and control panes were identical in glass type, dimensions, and construction, differing only in whether the bird collision prevention pattern was present. All glass panes measured 50 cm × 100 cm, and the pattern configuration of each sample was maintained consistently under both transparent and reflective conditions. Each sample was evaluated under both conditions using the same experimental procedure.

### 2.4. Experimental Birds

Wild birds were captured under permits issued by the Ongjin County Office, Incheon, Republic of Korea (Permit Nos. 25031801 for 2025 and 2026031701 for 2026), in accordance with the Wildlife Protection and Management Act of the Republic of Korea. The experimental procedures carried out in 2026 were approved by the Institutional Animal Care and Use Committee (IACUC) of Incheon National University (Approval No. INU-ANIM-2026-0014; 14 April 2026). The 2025 trials were not included within the scope of this IACUC approval. Bird capture was conducted during the spring migration period (April–June), from morning until before sunset. Wild birds were captured using mist nets (mesh size: 16 mm × 16 mm; Ecotone), which were checked at approximately 30 min intervals. The study was limited to small passerines that could be readily captured and safely tested in the flight tunnel. Birds weighing less than approximately 8 g or 30 g or more were excluded because of the potential risk of passing through the safety net or colliding with the glass. Species that typically move along the ground rather than by flight, or primarily locomote by climbing tree trunks or branches, were excluded because their characteristic locomotor behavior was incompatible with the flight-based choice test conducted in the tunnel. Following capture, each bird was visually assessed for its physical condition before testing. Individuals exhibiting signs of exhaustion or weakness were excluded from the experiment and released without undergoing testing. Captured birds were individually held in cloth bags, and bill length, wing length, tail length, tarsus length, body mass, and total length were measured before the flight test. To prevent repeated testing of the same individuals, each bird was fitted with a color identification band on the left leg before testing. Birds were released immediately at the capture site after completion of the test. If a previously banded bird was recaptured, it was released immediately without further testing. Eligible birds were tested sequentially as they were captured, without stratification by species. Age was not systematically categorized because the test aimed to evaluate the relative avoidance of treated versus untreated glass under standardized conditions, rather than to assess susceptibility to collision based on age. A total of 507 birds were tested, including 249 individuals under transparent conditions in 2025 and 258 under reflective conditions in 2026. The test birds consisted of small passerines, including species from the families Paridae, Emberizidae, and Muscicapidae (Table 1).

### 2.5. Statistical Analysis

All statistical analyses were conducted using R version 4.5.2 (R Foundation for Statistical Computing, Vienna, Austria). For each sample under each test condition, an exact binomial test was conducted to determine whether the observed avoidance response deviated from an expected choice probability of 0.5. Avoidance responses for the same sample under both transparent and reflective conditions were compared using Fisher’s exact test, and odds ratios (ORs) with 95% confidence intervals (CIs) were computed. The Holm method was employed to adjust for multiple comparisons among the sampless. The overall effect of the test condition on avoidance responses was examined using a binomial generalized linear model (GLM). The base model incorporated test condition and sample as explanatory variables. An additional model incorporating the interaction between test condition and sample (condition × sample) was fitted to assess whether the effect of test condition varied across samples. Models were compared using likelihood ratio tests (LRTs). The occurrence of invalid flights was analyzed using a binomial generalized linear model (GLM) to assess the effects of test condition and sample, as well as their interaction. To evaluate whether species affected avoidance responses, Pearson’s chi-square test (χ^2^ test) was performed for the major species with at least 37 valid flights. The effect of species was further evaluated by comparing the base GLM with a model additionally including species as an explanatory variable using a likelihood ratio test (LRT). The 95% confidence intervals for all proportions were calculated using the Wilson method, and statistical significance was set at *p* < 0.05 for all tests. The 70% avoidance rate referenced in this study corresponds to a tunnel score of 70 and, conversely, to a material threat factor (TF) of 30, where TF represents the percentage of valid flights directed toward the test material [22]. This value was used as a descriptive reference benchmark rather than as a statistical classification threshold. To evaluate the sensitivity of the study design to differences between test conditions, the minimum detectable effect (MDE) for the condition effect in the binomial generalized linear model was estimated using the observed cell sizes, with a two-sided significance level of 0.05 and 80% statistical power.

## 3. Results

### 3.1. Avoidance Performance Under Transparent and Reflective Conditions

**Figure 4:**
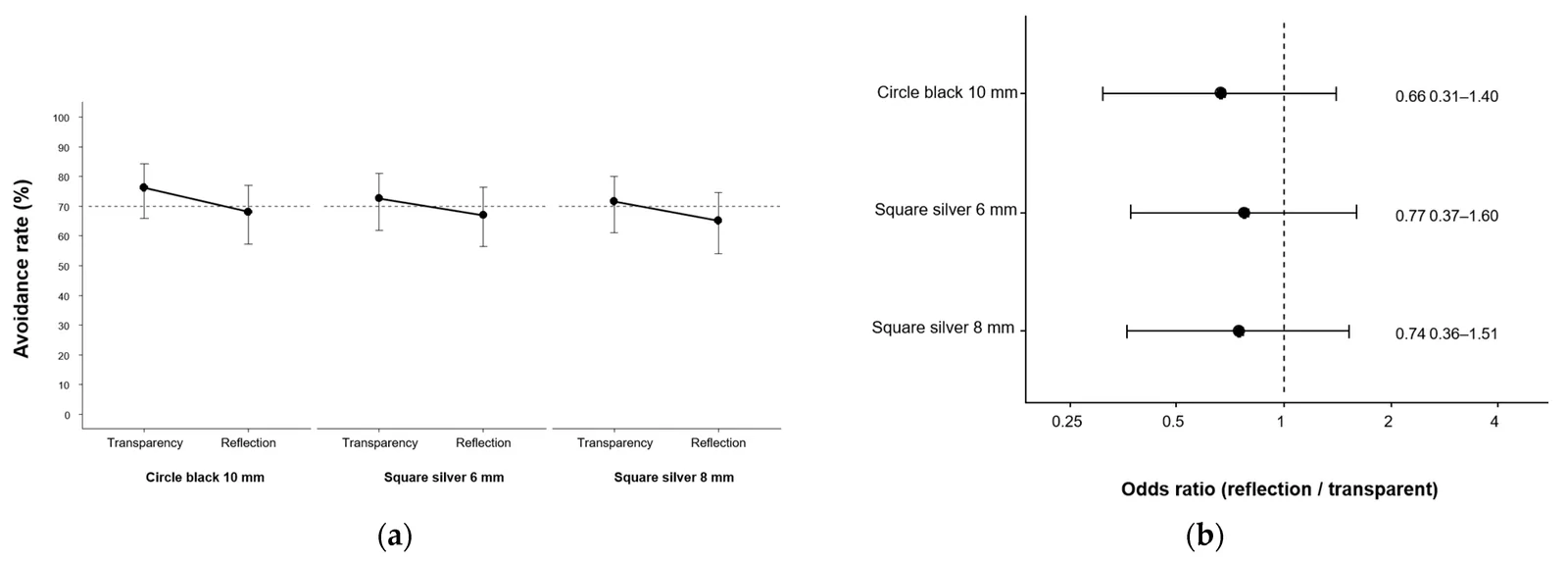
Comparison of avoidance performance across samples under transparent and reflective conditions. (**a**) Observed avoidance rates and Wilson 95% confidence intervals for the three samples. The dashed line represents the 70% avoidance rate criterion. (**b**) odds ratios (ORs) for avoidance in the reflective condition compared to the transparent condition, accompanied by 95% confidence intervals. The vertical dashed line indicates OR = 1.

All three samples showed lower avoidance rates under the reflective condition than under the transparent condition (Figure 4a). The avoidance rate for Sample a (10 mm black circle) decreased by 8.35 percentage points, from 76.25% to 67.90%. For Sample b (8 mm silver square), the avoidance rate decreased by 6.43 percentage points, from 71.43% to 65.00%, while that for Sample c (6 mm curved silver square) decreased by 5.43 percentage points, from 72.50% to 67.07%. Exact binomial tests showed that, in all six tests, the proportion of birds selecting the control glass was significantly higher than the 0.5 probability expected under equal choice (Table 2).

Fisher’s exact tests comparing avoidance responses between transparent and reflective conditions for each sample showed lower odds of avoidance under the reflective condition for all three samples (Figure 4b); however, none of the differences were statistically significant. The odds ratio (OR) was 0.661 (95% CI: 0.309–1.395, *p* = 0.293) for the 10 mm black circular pattern, 0.774 (95% CI: 0.372–1.597, *p* = 0.496) for the 6 mm curved silver square pattern, and 0.744 (95% CI: 0.364–1.514, *p* = 0.405) for the 8 mm silver square pattern. After adjustment for multiple comparisons using the Holm method, none of the comparisons remained statistically significant (adjusted *p* = 0.878).

### 3.2. Overall Effects of Transparent and Reflective Conditions and Their Interaction with Product

**Figure 5:**
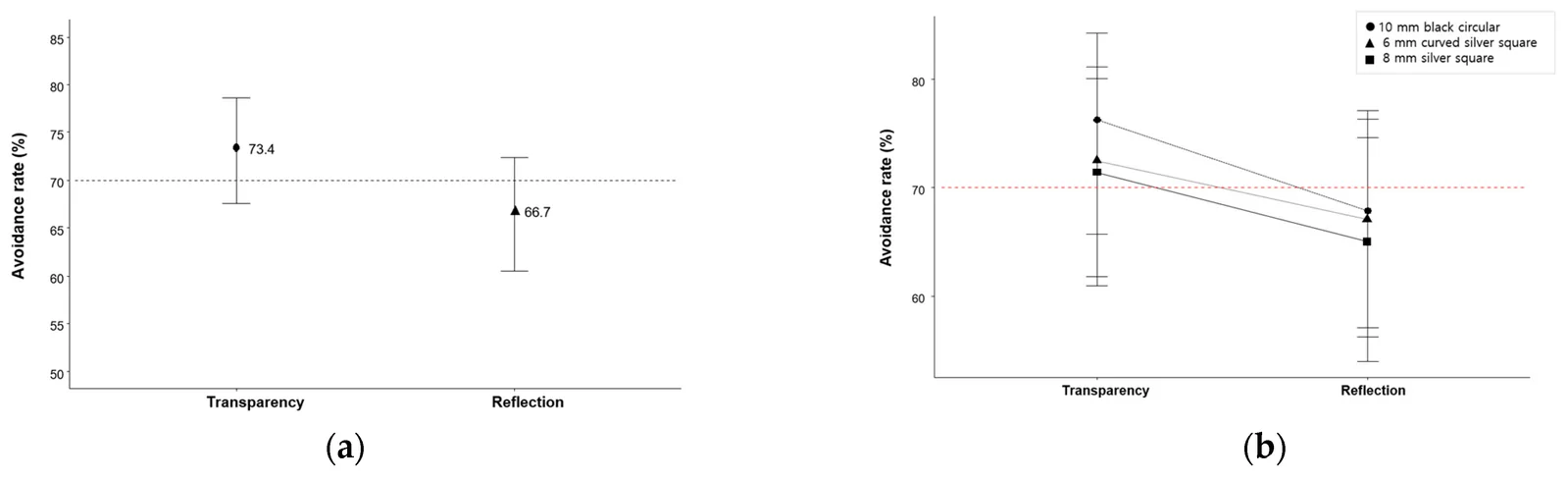
Estimated avoidance probabilities under transparent and reflective conditions based on the binomial logistic regression model (GLM). (**a**) Model-adjusted avoidance probabilities with 95% confidence intervals for each test condition. The dashed line indicates the 70% avoidance-rate criterion. (**b**) Estimated avoidance probabilities with 95% confidence intervals for each sample. Circles indicate the 10 mm black circular pattern, triangles the 6 mm curved silver square pattern, and squares the 8 mm silver square pattern.

The binomial logistic regression model (GLM), which accounted for differences among samples, estimated a lower probability of avoidance under the reflective condition than under the transparent condition; however, the effect of test condition was not statistically significant (OR = 0.725, 95% CI: 0.490–1.069, *p* = 0.105; Figure 5a). The model-adjusted mean avoidance rate was estimated at 73.42% (95% CI: 67.52–78.58%) under the transparent condition and 66.68% (95% CI: 60.51–72.33%) under the reflective condition, corresponding to a decrease of approximately 6.7 percentage points. When the same samples were compared between the two test conditions, all three showed a consistent trend toward lower estimated avoidance probabilities under the reflective condition (Figure 5b). The interaction between test condition and sample was not statistically significant (χ^2^ = 0.113, df = 2, *p* = 0.945). The MDE analysis indicated that the current design offered 80% power to detect a difference of approximately 12 percentage points between test conditions, which exceeded the observed model-adjusted difference of approximately 6.7%.

### 3.3. Comparison of Invalid Flight Rates Between Transparent and Reflective Conditions

**Figure 6:**
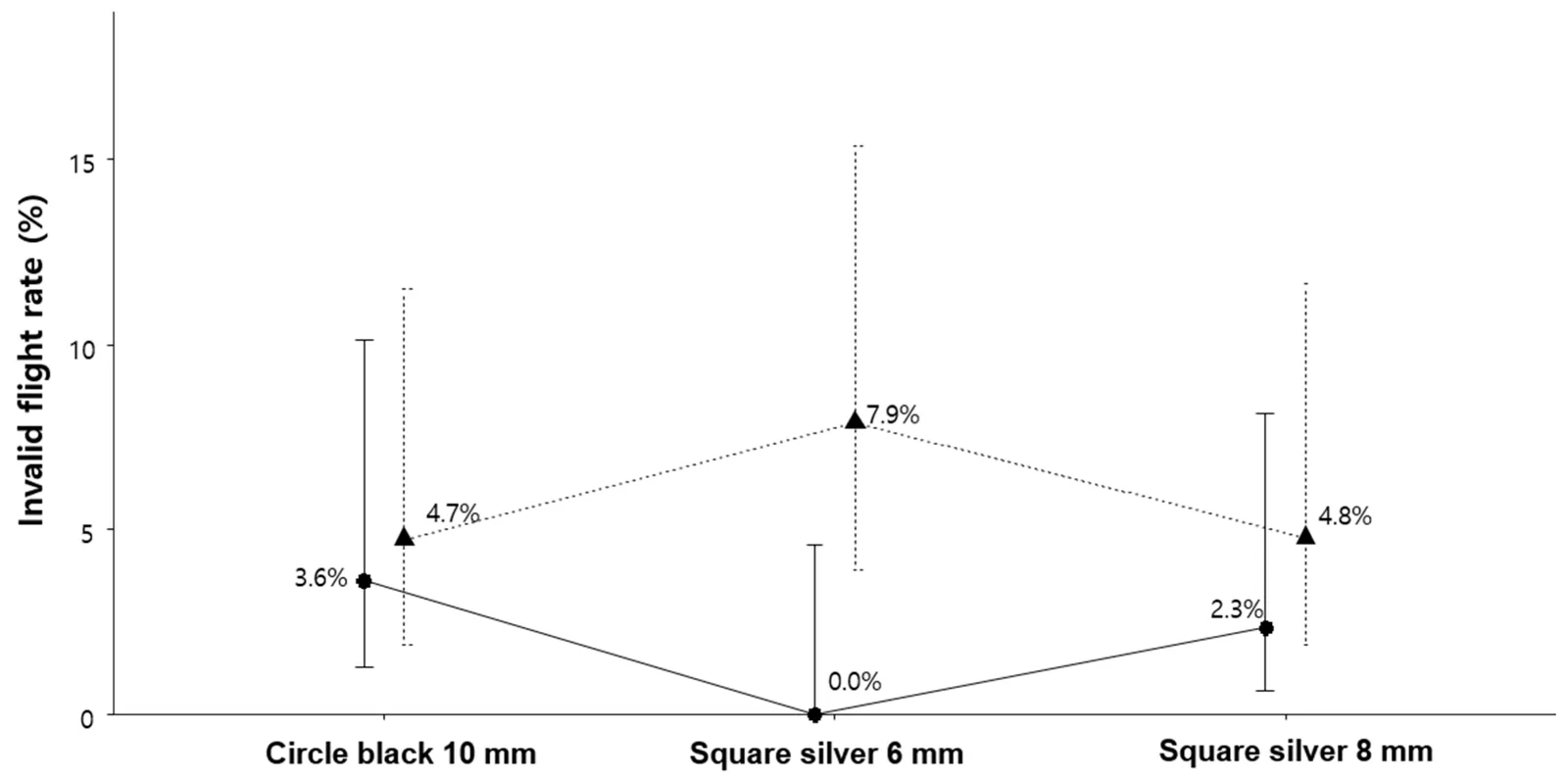
Invalid flight rates for each sample under transparent and reflective conditions. Error bars represent 95% confidence intervals calculated using the Wilson method. Circles and triangles indicate the transparent and reflective conditions, respectively.

All three samples exhibited higher rates of invalid flights under the reflective condition compared to the transparent condition (Figure 6). The invalid flight rate increased from 3.61% to 4.71% for the 10 mm black circular pattern, from 0.00% to 7.87% for the 6 mm silver square pattern, and from 2.33% to 4.76% for the 8 mm silver square pattern. Fisher’s exact tests conducted on individual samples revealed a significant difference solely for the 6 mm silver square pattern (*p* = 0.0146). In the binomial logistic regression model (GLM) controlling for sample effects, the test condition significantly influenced the occurrence of invalid flights (likelihood ratio test: χ^2^ = 5.05, df = 1, *p* = 0.025). The odds ratio (OR) for the reflective condition was 3.01, indicating a higher likelihood of an invalid flight compared to the transparent condition. The interaction between test condition and sample did not reach statistical significance (χ^2^ = 5.08, df = 2, *p* = 0.079).

### 3.4. Influence of Species Composition on Avoidance Responses

The observed avoidance rates of the six most frequently tested species (≥37 valid flights) were similar, ranging from 68.75% to 74.36%, with no significant association between species and avoidance response (χ^2^ = 0.491, df = 5, *p* = 0.992). A likelihood ratio test comparing the base GLM with a model additionally including species as a covariate showed that the inclusion of species did not significantly improve model fit (χ^2^ = 0.919, df = 5, *p* = 0.969). In the species-adjusted model, the odds ratio (OR) for the reflective condition was 0.710 (95% CI: 0.405–1.247, *p* = 0.234), compared with 0.725 (95% CI: 0.490–1.069, *p* = 0.105) in the base model.

## 4. Discussion

This study assessed whether the effectiveness of bird collision prevention patterned samples varies according to the optical conditions of the glass. To this end, we compared bird avoidance responses to the same patterned samples under two distinct test conditions: a transparent condition, in which an open background or sky image was positioned behind the glass [15,20], and a reflective condition, in which the sky was reflected on the glass surface. The avoidance rate was calculated based on the proportion of birds that selected the untreated control glass rather than the patterned test glass; thus, a higher avoidance rate indicated that birds recognized the pattern and avoided the treated surface. All three bird collision prevention samples demonstrated high avoidance rates under transparent conditions. Specifically, the point estimates of avoidance rates ranged from 71.43% to 76.25%, exceeding the 70% reference benchmark associated with a threat factor of 30 [15,16,23]; however, the 95% confidence intervals for all three patterned samples included 70%. It should be noted, however, that this threshold reflects a relative response observed under controlled tunnel test conditions rather than the actual reduction in bird collisions with buildings. When the same samples were tested under reflective conditions, avoidance rates for all three samples decreased by approximately 5–8 percentage points compared with those under transparent conditions, and the model-adjusted mean avoidance rate decreased by approximately 6.7 percentage points. The odds ratios for all three samples were less than 1; however, neither the individual comparisons nor the overall effect of the test condition in the generalized linear model reached statistical significance. Therefore, the current findings do not provide adequate evidence to conclude that reflective conditions generally reduce the effectiveness of bird collision prevention products. Nevertheless, the consistent direction of change observed across all three samples, along with a similar trend following adjustment for product effects, indicates that the impact of reflective conditions merits further investigation. In the current study design, the minimum detectable difference at 80% statistical power was approximately 12 percentage points, while the observed model-adjusted difference was approximately 6.7 percentage points. Therefore, the observed difference was smaller than the effect size that the current design was adequately powered to detect. This limitation, together with the confidence interval for the condition effect encompassing an odds ratio (OR) of 1, suggests that the observed decrease should be regarded as an estimated trend rather than conclusive evidence of a difference between test conditions. Current practices in the biological sciences increasingly prioritize interpreting effect sizes and confidence intervals over relying solely on binary significance testing [24,25]. These findings indicate that avoidance responses may differ between transparent and reflective test configurations, underscoring the necessity for further assessment across a wider spectrum of optical conditions [8].

The observed reduction in avoidance rates under reflective conditions suggests that reflections on glass surfaces may influence avian flight responses [21]. Birds may perceive the space visible through transparent glass, or the sky and vegetation reflected on the glass surface, as navigable airspace rather than recognizing the glass as a physical barrier [26,27]. Accordingly, collision risk may increase where glass surfaces reflect surrounding sky or vegetation [28]. Strong reflections may also interfere with the visual information provided by collision-reduction patterns, potentially reducing their effectiveness. The effectiveness of these patterns depends not only on spacing but also on visual characteristics such as size, arrangement, and contrast [15]. In this study, under the transparent condition, the patterns were presented on the glass surface while the background was visible behind the glass. Under the reflective condition, however, the patterns and the reflected sky were presented on the same glass surface. This visual overlap may have reduced the contrast between the patterns and the reflected background or made the patterns less visually distinct, potentially affecting the extent to which birds perceived them as markings indicating the presence of a physical barrier [15,29]. Because visual sensitivity varies across wavelengths and among bird species, the perceptual contrast of a prevention pattern may differ from estimates based solely on human vision. These findings suggest that pattern design, pattern placement on the glass, and surrounding reflections may collectively influence the visual information available to birds. However, because both pattern design and installation method varied among the products tested in this study, the contribution of each factor to the observed differences under reflective conditions could not be determined independently. Future studies should control these factors separately to distinguish the effects of reflection and installation method and further examine how each factor influences avian avoidance responses.

The analysis of invalid flights, conducted separately from the avoidance rate analysis, suggests that reflective conditions may affect not only the final directional choice but also flight behavior during the approach. Under reflective conditions, the odds of an invalid flight were approximately three times higher than under transparent conditions (OR = 3.01; χ^2^ = 5.05, df = 1, *p* = 0.025), and this increase was observed across all three patterned samples tested. Invalid flights included several behavioral responses, such as failure to initiate flight, cessation of flight before reaching the end of the tunnel, and repeated changes in direction near the safety net. However, since luminance was not quantified between the two test configurations, its potential contribution to the increased frequency of invalid flights under the reflective condition cannot be ruled out. The higher occurrence of invalid flights under reflective conditions therefore suggests that reflections may influence flight behavior during the approach; however, because these behaviors were combined into a single binary category, the underlying behavioral mechanism cannot be determined from the present data [11]. Although a reflected sky background may act as a strong visual cue and promote more direct flight, the simultaneous presentation of patterns and reflections may create conflicting visual information, potentially resulting in delayed approaches or changes in flight direction. However, because this study combined multiple behavioral responses into a single binary category of invalid flight, the observed increase under reflective conditions cannot be attributed to a specific behavioral mechanism. The variation in the occurrence of invalid flights across test conditions also suggests that evaluation based solely on avoidance rates may not fully capture the range of behavioral responses occurring during tunnel tests. Future studies should therefore classify invalid flights by behavioral type alongside avoidance rates and incorporate analyses of flight trajectories and approach behavior [11,21]. Such analyses could provide a more detailed understanding of how reflective conditions influence avian flight responses and behavioral decisions during approach.

Species composition did not appear to substantially influence the effect of test condition observed in this study. Avoidance rates were similar among the primary species, and including species as a covariate did not significantly improve model fit compared with the baseline model. In addition, the effect of the reflective condition estimated from the species-adjusted model (OR = 0.710) was similar to that estimated from the baseline model (OR = 0.725). These results indicate that species composition, especially the responses of the dominant species tested, did not significantly influence the observed trend of reduced avoidance rates under reflective conditions within the current tunnel-testing protocol. The single-exposure design also minimized potential learning effects and repeated measurements of the same individuals. The tunnel test system was designed to offer a standardized evaluation of the relative effectiveness of bird collision prevention products, rather than to assess species-specific collision responses. The use of small passerines aligns with established tunnel-testing protocols, whose results have been regarded as broadly applicable across passerine species [15]. The selection of small passerines also reflects the structural and safety requirements of the indoor tunnel system, which was designed for birds capable of completing directional flights within the experimental setting [23]. Because bird collisions occur across a wide range of taxa, extending this framework to groups with different body sizes, visual fields, and flight characteristics, such as raptors and waterbirds, through appropriately adapted test configurations would further broaden its applicability [18,19].

The reflective testing environment developed in this study was designed to simulate a reflected sky background on the glass surface under controlled conditions. This approach allowed for the comparison of bird responses to prevention patterns between a condition in which the visual background was visible through the glass and one in which it appeared as a reflection on the glass surface. The reflective configuration required alterations in glass orientation and background arrangement to generate the reflected sky image, consistent with prior flight experiments in which glass orientation was modified to display the reflected sky to approaching birds [16]. However, the flight distance was maintained consistently across both conditions, as flights were terminated by the safety mist net positioned in front of the test panes in each configuration. These structural differences were necessary to represent the prevention patterns against a reflected visual background, rather than to alter the flight distance. Nevertheless, differences in viewing geometry and luminance between the two configurations cannot be excluded [21]. Luminance was not measured during the trials; consequently, its potential influence on flight motivation and the occurrence of invalid flights remains uncertain. The visible and ultraviolet reflectance of the glass was also not quantified. Therefore, the reflective configuration should not be regarded as indicative of a particular reflectance level or category of architectural glazing. Accordingly, the observed differences should be interpreted as responses to the transparent and reflective test configurations rather than as the isolated effect of reflection. The preliminary test utilizing identical untreated glass panes revealed no significant left–right preference, and the same configuration of treated and control panes was preserved across both conditions. However, potential positional effects were not independently evaluated because pane positions remained fixed throughout testing. Further research incorporating varied pane positions and systematic measurements of luminance and other optical properties would facilitate a more accurate evaluation of avoidance responses across diverse test configurations. Conventional tunnel tests are valuable for assessing the relative avoidance performance of bird collision prevention products under standardized conditions [15]. However, glass surfaces in actual built environments often reflect the surrounding sky and vegetation, which have been associated with an increased risk of bird collisions [1,28]. In this study, the point estimates of avoidance rates were lower under the reflective condition for all three patterned samples, although the confidence intervals for both conditions overlapped the 70% criterion. In addition, the increased occurrence of invalid flights under reflective conditions suggests that the optical conditions of glass may influence the outcomes of product evaluations. Therefore, to better evaluate the applicability of bird collision prevention products to actual built environments, future assessment frameworks should consider reflective conditions alongside existing transparency-based tests. Rather than replacing conventional transparency-based methods, reflective testing could serve as a complementary approach for evaluating product performance under a broader range of optical conditions [13]. Future studies should include glass with different optical properties and a wider range of bird collision prevention products, together with field validation on actual buildings, to assess the applicability of the reflective testing method developed in this study [23]. These efforts could contribute to the development of evaluation methods that better account for the range of conditions encountered by birds in actual built environments.

## 5. Conclusions

This study evaluated avian avoidance responses to collision-deterrent products using a flight tunnel test that simulated reflections on glass surfaces, with the results compared with those under transparent conditions. Although lower avoidance rates were observed for all three patterned samples under the reflective condition, the overall effect of test condition on avoidance was not statistically significant. The occurrence of invalid flights, however, increased significantly under the reflective condition. These findings suggest that incorporating reflective conditions may provide additional information when evaluating bird collision prevention products beyond that obtained from transparency-based tests alone. The reflective testing protocol developed in this study may therefore complement existing transparency-based tunnel tests, although further evaluation using larger sample sizes and a broader range of glass and optical conditions is needed.

## Figures and Tables

**Table 1:** Number of birds used in flight tunnel experiments.

Species		Individuals	%
Great Tit	*Parus minor*	128	25.2
Red-flanked Bluetail	*Tarsiger cyanurus*	75	14.8
Brambling	*Fringilla montifringilla*	48	9.5
Warbling White-eye	*Zosterops japonicus*	41	8.1
Yellow-throated Bunting	*Emberiza elegans*	40	7.9
Coal Tit	*Periparus ater*	37	7.3
Daurian Redstart	*Phoenicurus auroreus*	23	4.5
Yellow-bellied Tit	*Pardaliparus venustulus*	17	3.4
Black-faced Bunting	*Emberiza spodocephala*	15	3.0
Rufous-tailed Robin	*Larvivora akahige*	13	2.6
Eurasian Siskin	*Spinus spinus*	11	2.2
Others (19 species)		59	11.6
Total		507	100

**Table 2:** Avoidance rates and exact binomial test results for each sample under transparent and reflective conditions.

Test	Sample	Total Flights	Invalid Flight	Valid Flight	Flights to Control	Flights to Sample	Avoidance Rate (%)	Score	*p*
Transparent	Black circle, 10 mm	83	3	80	61	19	76.25	76	<0.001
Transparent	Silver square, 8 mm	86	2	84	60	24	71.43	71	<0.001
Transparent	Silver square, 6 mm (curved)	80	0	80	58	22	72.50	73	<0.001
Reflective	Black circle, 10 mm	85	4	81	55	26	67.90	68	0.002
Reflective	Silver square, 8 mm	84	4	80	52	28	65.00	65	0.010
Reflective	Silver square, 6 mm (curved)	89	7	82	55	27	67.07	67	0.003

## Data Availability

The data presented in this study are available upon request from the corresponding author.
